# The ethics of using artificial intelligence in scientific research: new guidance needed for a new tool

**DOI:** 10.1007/s43681-024-00493-8

**Published:** 2024-05-27

**Authors:** David B. Resnik, Mohammad Hosseini

**Affiliations:** 1 National Institute of Environmental Health Sciences, Durham, USA; 2 Department of Preventive Medicine, Northwestern University Feinberg School of Medicine, Chicago, IL USA; 3 Galter Health Sciences Library and Learning Center, Northwestern University Feinberg School of Medicine, Chicago, IL USA

**Keywords:** Artificial intelligence, Ethics, Research, Trust, Transparency, Accountability, Bias, Error, Explainability, Social responsibility

## Abstract

Using artificial intelligence (AI) in research offers many important benefits for science and society but also creates novel and complex ethical issues. While these ethical issues do not necessitate changing established ethical norms of science, they require the scientific community to develop new guidance for the appropriate use of AI. In this article, we briefly introduce AI and explain how it can be used in research, examine some of the ethical issues raised when using it, and offer nine recommendations for responsible use, including: (1) Researchers are responsible for identifying, describing, reducing, and controlling AI-related biases and random errors; (2) Researchers should disclose, describe, and explain their use of AI in research, including its limitations, in language that can be understood by non-experts; (3) Researchers should engage with impacted communities, populations, and other stakeholders concerning the use of AI in research to obtain their advice and assistance and address their interests and concerns, such as issues related to bias; (4) Researchers who use synthetic data should (a) indicate which parts of the data are synthetic; (b) clearly label the synthetic data; (c) describe how the data were generated; and (d) explain how and why the data were used; (5) AI systems should not be named as authors, inventors, or copyright holders but their contributions to research should be disclosed and described; (6) Education and mentoring in responsible conduct of research should include discussion of ethical use of AI.

## Introduction: exponential growth in the use of artificial intelligence in scientific research

In just a few years, artificial intelligence (AI) has taken the world of scientific research by storm. AI tools have been used to perform or augment a variety of scientific tasks, including^1^:

- Analyzing data and images [34, 43, 65, 88, 106, 115, 122, 124, 149, 161].
- Interpreting data and images [13, 14, 21, 41].
- Generating hypotheses [32, 37, 41, 107, 149].
- Modelling complex phenomena [32, 41, 43, 122, 129].
- Designing molecules and materials [15, 37, 43, 205].
- Generating data for use in validation of hypotheses and models [50, 200].
- Searching and reviewing the scientific literature [30, 72].
- Writing and editing scientific papers, grant proposals, consent forms, and institutional review board applications [3, 53, 54, 82, 163].
- Reviewing scientific papers and other research outputs [53, 54, 98, 178, 212].

The applications of AI in scientific research appears to be limitless, and in the next decade AI is likely to completely transform the process of scientific discovery and innovation [6–9, 105, 201].

Although using AI in scientific research has steadily grown, ethical guidance has lagged far behind. With the exception of using AI to draft or edit scientific papers (see discussion in Sect. 7.6), most codes and policies do not explicitly address ethical issues related to using AI in scientific research. For example, the 2023 revision of the European Code of Conduct for Research Integrity [4] briefly discusses the importance of transparency. The code stipulates that researchers should report “their results and methods including the use of external services or AI and automated tools” (Ibid., p. 7) and considers “hiding the use of AI or automated tools in the creation of content or drafting of publications” as a violation of research integrity (Ibid. p. 10). One of the most thorough and up-to-date institutional documents, the National Institutes of Health Guidelines and Policies for the Conduct of Research provides guidance for using AI to write and edit manuscripts but not for other tasks [158].^2^ Codes of AI ethics, such as UNESCO’s [223] Ethics of Artificial Intelligence and the Office of Science and Technology Policy’s [168, 169] Blueprint for an AI Bill of Rights, provide useful guidance for the development and use of AI in general without including specific guidance concerning the development and use of AI in scientific research [215].

There is therefore a gap in ethical and policy guidance concerning AI use in scientific research that needs to be filled to promote its appropriate use. Moreover, the need for guidance is urgent because using AI raises novel epistemological and ethical issues related to objectivity, reproducibility, transparency, accountability, responsibility, and trust in science [9, 102]. In this paper, we will examine important questions related to AI’s impact on ethics of science. We will argue that while the use of AI does not require a radical change in the ethical norms of science, it will require the scientific community to develop new guidance for the appropriate use of AI. To defend this thesis, we will provide an overview of AI and an account of ethical norms of science, and then we will discuss the implications of AI for ethical norms of science and offer recommendations for its appropriate use.

## What is AI?

AI can be defined as “a technical and scientific field devoted to the engineered system that generates outputs such as content, forecasts, recommendations or decisions for a given set of human-defined objectives [114].” AI is a *subfield* within the discipline of computer science [144]. However, the term ‘AI’ is also commonly used to refer to *technologies (or tools)* that can perform human tasks that require intelligence, such as perception, judgment, reasoning, or decision-making. We will use both senses of ‘AI’ in this paper, depending on the context.

While electronic calculators, cell phone apps, and programs that run on personal computers can perform functions associated with intelligence, they are not generally considered to be AI because they do not “learn” from the data [108]. As discussed below, AI systems can learn from the data insofar as they can adapt their programming in response to input data. While applying the term ‘learning’ to a machine may seem misleadingly anthropomorphic, it does make sense to say that a machine can learn if learning is regarded as a change in response to information about the environment [151]. Many different entities can learn in this sense of the term, including the immune system, which changes after being exposed to molecular information about pathogens, foreign objects, and other things that provoke an immune response.

**Fig. 1:**
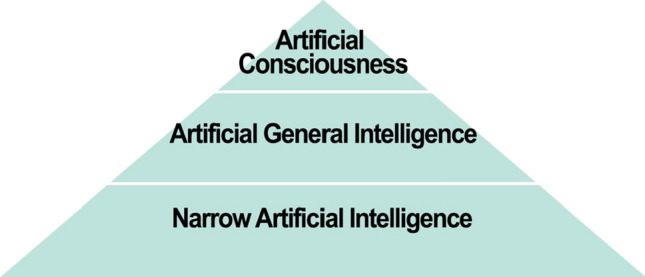
Levels of Artificial Intelligence, according to Turing [219]

This paper will focus on what is commonly referred to as narrow (or weak) AI, which is already being extensively used in science. Narrow AI has been designed and developed to do a specific task, such as playing chess, modelling complex phenomena, or identifying possible brain tumors in diagnostic images [151]. See Fig. 1.^3^ Other types of AI discussed in the literature include broad AI (also known as artificial general intelligence or AGI), which is a machine than can perform multiple tasks requiring human-like intelligence; and artificial consciousness (AC), which is a form of AGI with characteristics widely considered to be essential for consciousness [162, 219]. Because there are significant technical and conceptual obstacles to developing AGI and AC, it may be years before machines have this degree of human-like intelligence [206, 227].^4^

## What is machine learning?

Machine learning (ML) can be defined as a branch of AI “that focuses on the using data and algorithms to enable AI to imitate the way that humans learn, gradually improving its accuracy [112].” There are several types of ML, including support vector machines, decisions trees, and neural networks. In this paper we will focus on ML that uses artificial neural networks (ANNs).

**Fig. 2:**
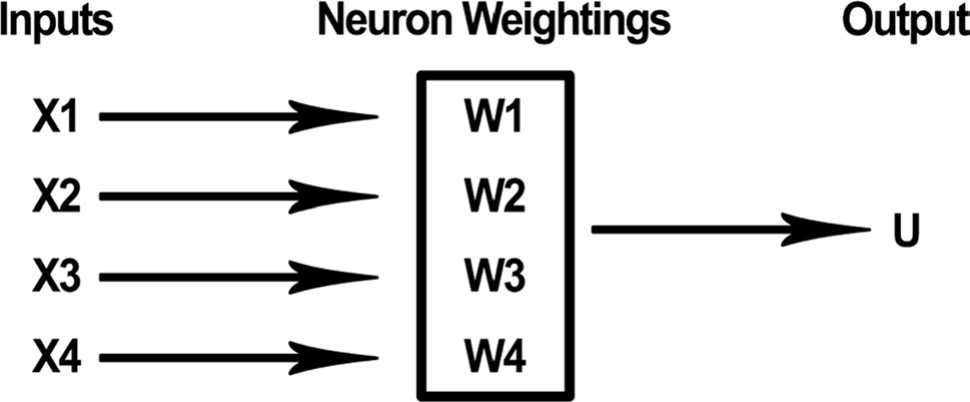
Artificial neuron

An ANN is composed of artificial neurons, which are modelled after biological neurons. An artificial neuron receives a series of computational inputs,^5^ applies a function, and produces an output. The inputs have different weightings. In most applications, a specific output is generated only when a certain threshold value for the inputs is reached. In the example below, an output of ‘1’ would be produced if the threshold is reached; otherwise, the output would be ‘0’. See Fig. 2. A pair statements describing how a very simple artificial neuron processes inputs could be as follows:

$$\begin{gathered} {\text{If }}\left[ {\left( {{\text{x1}}} \right)\left( {{\text{w1}}} \right) \, + \, \left( {{\text{x2}}} \right)\left( {{\text{w2}}} \right) \, + \, \left( {{\text{x3}}} \right)\left( {{\text{w3}}} \right) \, + \, \left( {{\text{x4}}} \right)\left( {{\text{w4}}} \right) \, > {\text{ T}}} \right],{\text{ then output U }} = { 1} \hfill \\ {\text{If }}\left[ {\left( {{\text{x1}}} \right)\left( {{\text{w1}}} \right) \, + \, \left( {{\text{x2}}} \right)\left( {{\text{w2}}} \right) \, + \, \left( {{\text{x3}}} \right)\left( {{\text{w3}}} \right) \, + \, \left( {{\text{x4}}} \right)\left( {{\text{w4}}} \right) \le {\text{T}}} \right],{\text{ then output U }} = \, 0 \hfill \\ \end{gathered}$$

where x1, x2, x3, and x4 are inputs; w1, w2, w3, and w4 are weightings, T is a threshold value; and U is an output value (1 or 0). An artificial neuron is represented schematically in Fig. 2, below.

**Fig. 3:**
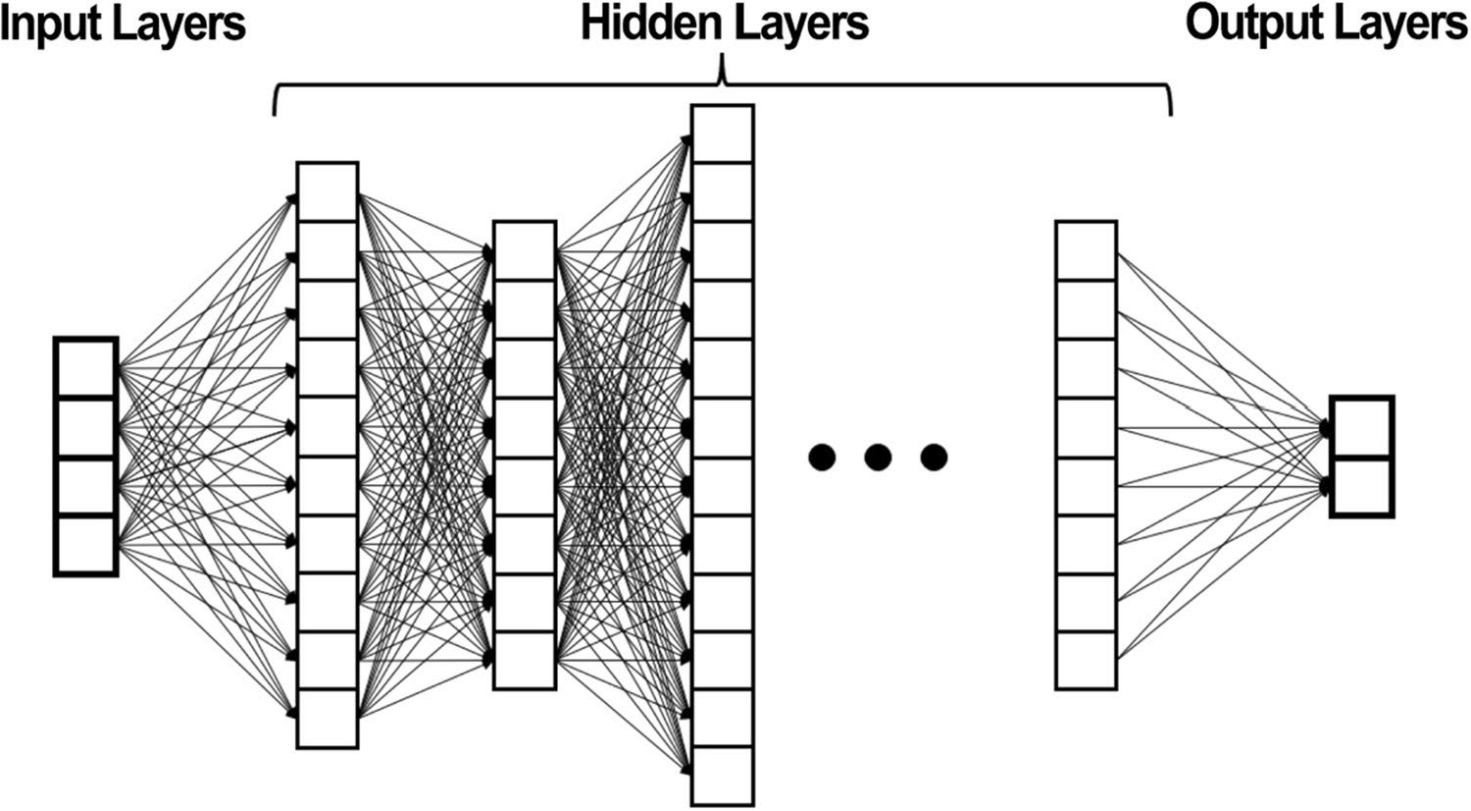
Deep learning artificial neural network [38]

A single neuron may have dozens of inputs. An ANN may consist of thousands of interconnected neurons. In a deep learning ANN, there may be many hidden layers of neurons between the input and output layers. See Fig. 3.

Training (or reinforcement) occurs when the weightings on inputs are changed in response to system’s output. Changes in the weightings are based on their contribution to the neuron’s error, which can be understood as the difference between the output value and the correct value as determined by the human trainers (see discussion of error in Sect. 5). Training can occur via supervised or unsupervised learning. In supervised learning, the ANN works with labelled data and becomes adept at correctly representing structures in the data recognized by human trainers. In unsupervised learning, the ANN works with unlabeled data and discovers structures inherent in the data that might not have been recognized by humans [59, 151]. For example, to use supervised learning to train an ANN to recognize dogs, human beings could present the system with various images and evaluate the accuracy of its output accordingly. If the ANN labels an image a “dog” that human beings recognize as a dog, then its output would be correct, otherwise, it would be incorrect (see discussion of error in Sects. 5.1 and 5.5). In unsupervised learning, the ANN would be presented with images and would be reinforced for accurately modelling structures inherent in the data, which may or may not correspond to patterns, properties, or relationships that humans would recognize or conceive of.

For an example of the disconnect between ML and human processing of information, consider research conducted by Roberts et al. [195]. In this study, researchers trained an ML system on radiologic images from hospital patients so that it would learn to identify patients with COVID-19 and predict the course of their illness. Since the patients who were sicker tended to laying down when their images were taken, the ML system identified laying down as a diagnostic criterion and disease predictor [195]. However, laying down is a confounding factor that has nothing to do with the likelihood of having COVID-19 or getting very sick from it [170]. The error occurred because the ML system did not account for this fundamental fact of clinical medicine.

Despite problems like the one discovered by Roberts et al. [195], the fact that ML systems process and analyze data differently from human beings can be a great benefit to science and society because these systems may be able to identify useful and innovative structures, properties, patterns, and relationships that human beings would not recognize. For example, ML systems have been able to design novel compounds and materials that human beings might not be able to conceive of [15]. That said, the disconnect between AI/ML and human information processing can also make it difficult to anticipate, understand, control, and reduce errors produced by ML systems. (See discussion of error in Sects. 5.1–5.5).

Training ANNs is a resource-intensive activity that involves gigabytes of data, thousands of computers, and hundreds of thousands of hours of human labor [182, 229]. A system can continue to learn after the initial training period as it processes new data [151]. ML systems can be applied to any dataset that has been properly prepared for manipulation by computer algorithms, including digital images, audio and video recordings, natural language, medical records, chemical formulas, electromagnetic radiation, business transactions, stock prices, and games [151].

**Fig. 4:**
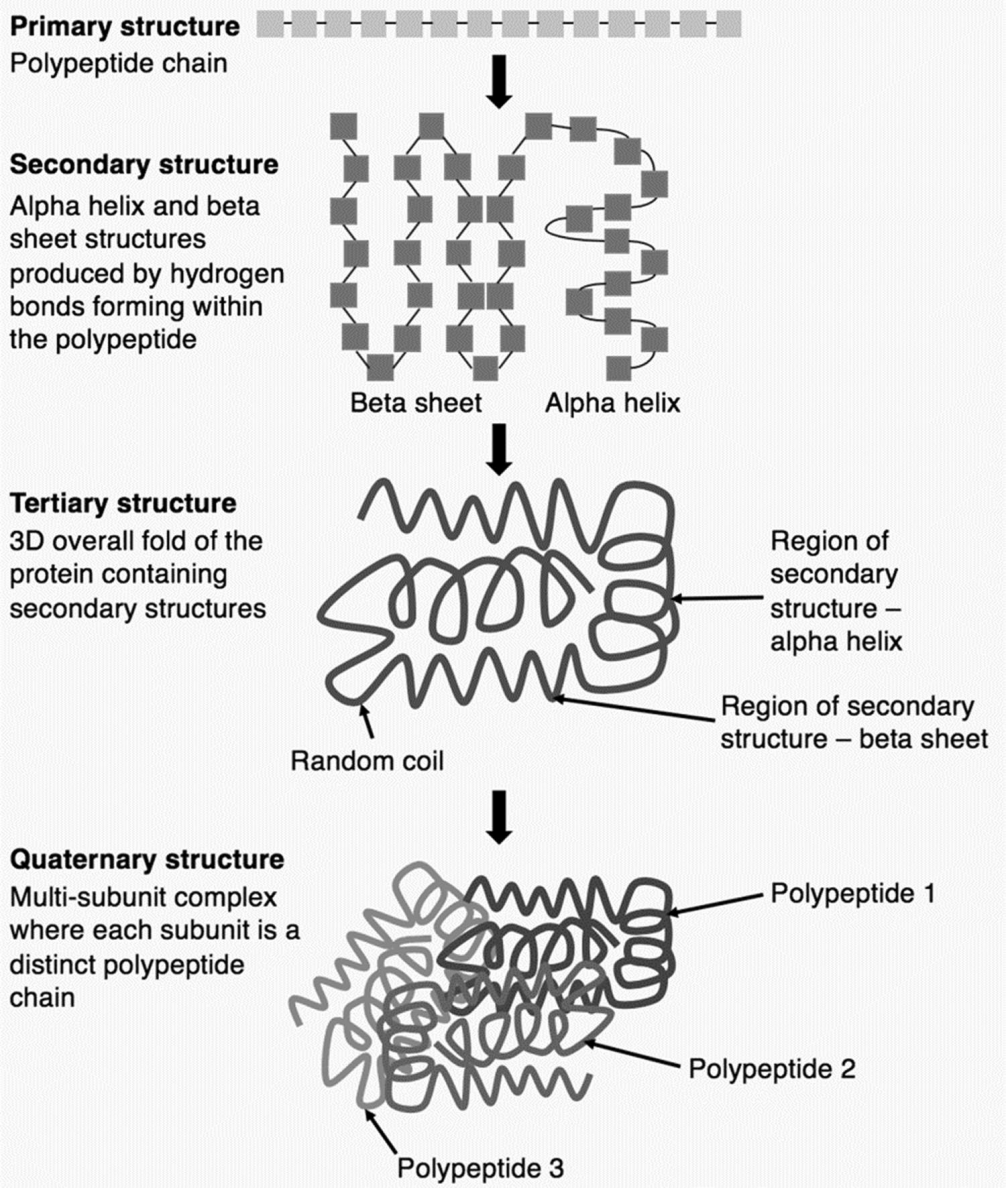
Protein folding. CC BY-SA 4.0 DEED [45]

One of the most impressive feats accomplished by ML systems is their contribution to solving the protein folding problem [41]. See Fig. 4. A protein is composed of one or more long chains of amino acids known as polypeptides. The three-dimensional (3-D) structure of the protein is produced by folding of the polypeptide(s), which is caused by the interplay of hydrogen bonds, Van der Waals attractive forces, and conformational entropy between different parts of the polypeptide [2]. Molecular biologists and biochemists have been trying to develop rules for predicting the 3-D structures of proteins from amino acid sequences since the 1960s, but this is, computationally speaking, a very hard problem, due to the immense number of possible ways that polypeptides can fold [52, 76]. Tremendous progress on the protein-folding problem was made in 2022, when scientists demonstrated that an ML system, DeepMind’s AlphaFold, can predict 3-D structures from amino acid sequences with 92.4% accuracy [118, 204]. AlphaFold, which built upon available knowledge of protein chemistry [176], was trained on thousands of amino acids sequences and their corresponding 3-D structures. Although human researchers still needed to test and refine AlphaFold’s output to ensure that the proposed structure is 100% accurate, the ML system greatly improves the efficiency of protein chemistry research [216]. Recently developed ML systems can generate new proteins by going in the opposite direction and predicting amino acids sequences from 3-D protein structures [156]. Since proteins play a key role in the structure and function of all living things, these advances in protein science are likely to have important applications in different areas of biology and medicine [204].

## What is generative AI?

Not only can ML image processing systems recognize patterns in the data that correspond to objects (e.g., cat, dog, car), when coupled with appropriate algorithms they can also generate images in response to visual or linguistic prompts [87]. The term ‘generative AI’ refers to “deep-learning models that can generate high-quality text, images, and other content based on the data they were trained on” [111].

**Fig. 5:**
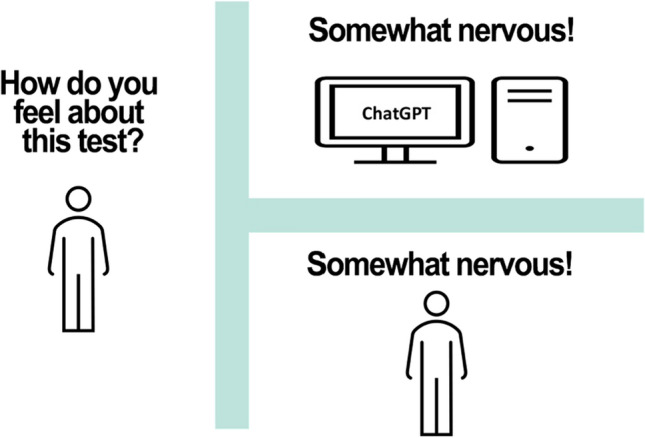
The Turing test. Computer scientist Alan Turing [220] proposed a famous test for determining whether a machine can think. The test involves a human interrogating another person, and a computer. The interrogator poses questions to the interviewees, who are in different rooms, so that interrogator cannot see where the answers are coming from. If the interrogator cannot distinguish between answers to questions given by another person and answers provided by a computer, then the computer passes the Turing test

Perhaps the most well-known types of generative AI are those that are based on large language models (LLMs), such as chatbots like OpenAI’s ChatGPT and Google’s Gemini, which analyze, paraphrase, edit, translate, and generate text, images and other types of content. LLMs are statistical algorithms trained on huge sets of natural language data, such as text from the internet, books, journal articles, and magazines. By processing this data, LLMs can learn to estimate probabilities associated with possible responses to text and can rank responses according to the probability that they will be judged to be correct by human beings [151]. In just a few years, some types of generative AI, such as ChatGPT, have become astonishingly proficient at responding to text data. ChatGPT has passed licensing exams for medicine and law and scored in the 93rd percentile on the Scholastic Aptitude Test reading exam and in the 89th percentile on the math exam [133, 138, 232]. Some researchers have used ChatGPT to write scientific papers and have even named them as authors [48, 53, 54, 167].^6^ Some LLMs are so adept at mimicking the type of discourse associated with conscious thought that computer scientists, philosophers, and cognitive psychologists are updating the Turing test (see Fig. 5) to more reliably distinguish between humans and machines [5, 22].

## Challenges of using AI

### Error

It has been long known that any AI systems are not error-free. To understand this topic, it is important to define ‘error’ and distinguish between systemic errors and random errors. The word ‘error’ has various meanings: we speak of grammatical errors, reasoning errors, typographical errors, measurement errors, etc. What these different senses of ‘error’ have in common is (1) errors involve divergence from a standard of correctness; and (2) errors, when committed by conscious beings, are unintentional; that is, they are accidents or mistakes and different from frauds, deceptions, or jokes.

If we set aside questions related to intent on the grounds that AI systems are not moral agents (see discussion in Sect. 7.6), *we can think of AI error as the difference between the output of an AI system and the correct output*. The difference between an AI output and the correct output can be measured quantitatively or qualitatively, depending on what is being measured and the purpose of the measurement [151]. For example, if a ML image recognition tool is presented with 50 images of wolves and 50 images of dogs, and it labels 98 of them correctly, we could measure its error quantitatively (i.e., 2%). In other cases, we might measure (or describe) error qualitatively. For example, if we ask ChatGPT to write a 12-line poem about a microwave oven in the style Edgar Allan Poe, we could rate its performance as ‘excellent,’ ‘very good,’ ‘good,’ ‘fair,’ or ‘poor.’ We could also assign numbers to these ratings to convert qualitative measurements into quantitative assessments (e.g., 5 = excellent, 4 = very good).

The correct output of an AI system is ultimately defined by its users and others who may be affected. For example, radiologists define correctness for reading diagnostic images; biochemists define the standard for modeling proteins; and attorneys, judges, clients, and law professors define the standard for writing legal briefs. In some contexts, such as testing hypotheses or reading radiologic images, ‘correct’ may mean ‘true’; in other contexts, such as generating text or creating models, it may simply mean ‘acceptable’ or ‘desirable.’^7^ While AI systems can play a key role in providing information that is used to define correct outputs (for example, when a system is used to discover new chemical compounds or solve complex math problems), human beings are ultimately responsible for determining whether outputs are correct (see discussion of moral agency in Sect. 7.6).

### Random versus systemic errors (*Bias*)

**Fig. 6:**
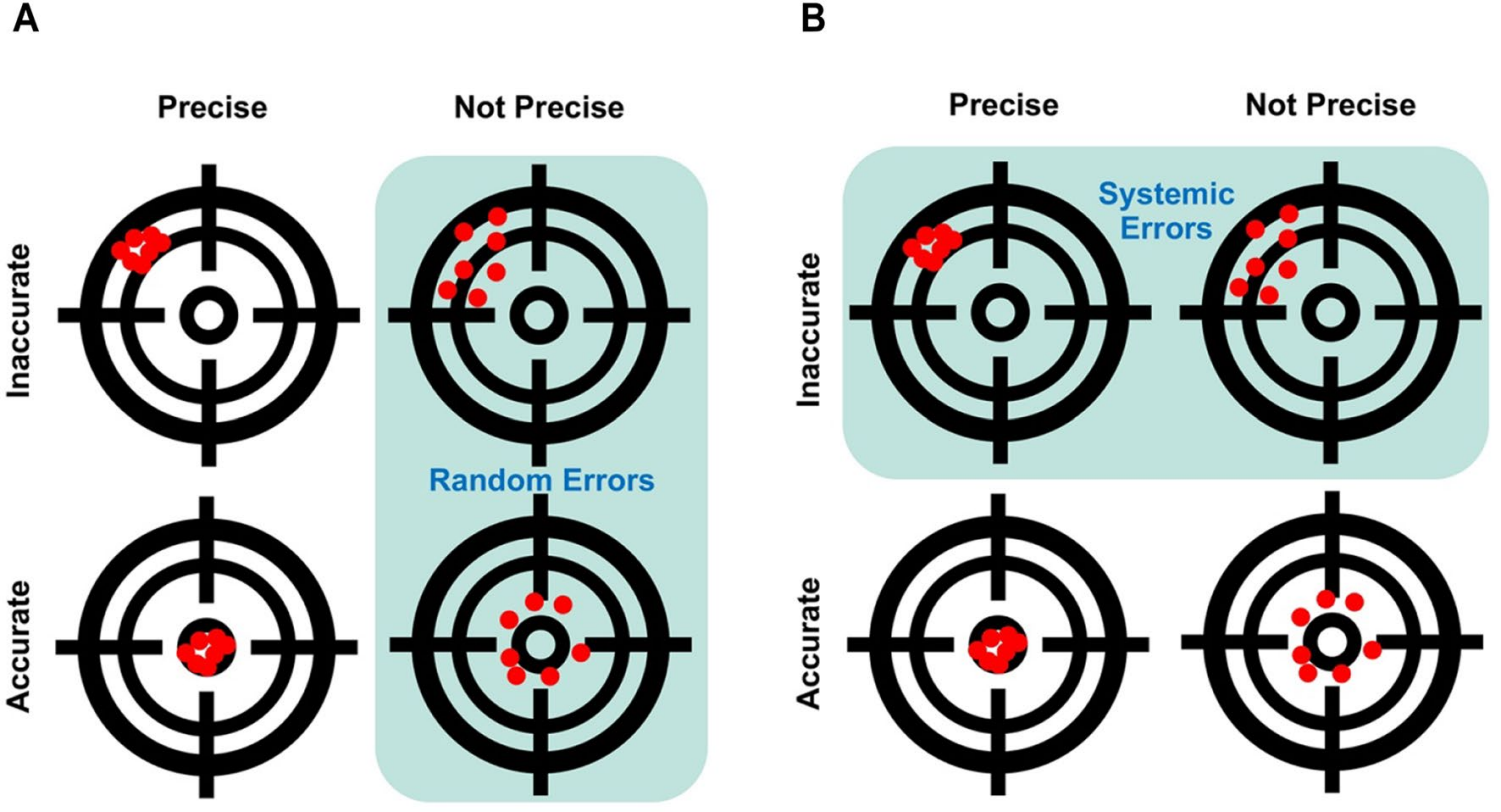
Random errors versus systemic errors

We can use an analogy with target shooting to think about the difference between random and systemic errors [94]. If error is understood as the distance of a bullet hole from a target, then random error would be a set of holes distributed randomly around the target without a discernable pattern (Fig. 6A), while systemic error (or bias) would be a set of holes with a discernable pattern, for example holes skewed in a particular direction (Fig. 6B). The accuracy of a set of bullet holes would be a function of their distance from the target, while their precision would be a function of their distance from each other [27, 172, 184].

The difference between systemic and random errors can be ambiguous because errors that appear to be random may be shown to be systemic when one acquires more information about how they were generated or once a pattern is discerned.^8^ Nevertheless, the distinction is useful. Systemic errors are often more detrimental to science and society than random ones, because they may negatively affect many different decisions involving people, projects, and paradigms. For example, racist biases distorted most research on human intelligence from the 1850s to the 1960s, including educational policies based on the applications of intelligence research. As will be discussed below, AI systems can make systemic and random errors [70, 174].

### AI biases

Since AI systems are designed to accurately represent the data on which they are trained, they can reproduce or even amplify racial, ethnic, gender, political, or other biases in the training data and subsequent data received [131]. The computer science maxim “garbage in, garbage out” applies here. Studies have shown that racial and ethnic biases impact the use of AI/ML in medical imaging, diagnosis, and prognosis due to biases in healthcare databases [78, 154]. Bias is also a problem in using AI systems to find relationships between genomics and disease due to racial and ethnic prejudices in genomic databases [55]. LLMs are also impacted by various biases inherent in their training data, and when used in generative AI models like ChatGPT, can propagate biases related to race, ethnicity, nationality, gender, sexuality, age, and politics [25, 171].^9^

Because scientific theories, hypotheses, and models are based on human perceptual categories, concepts, and assumptions, bias-free research is not possible [121, 125, 137]. Nevertheless, scientists can (and should) take steps to understand sources of bias and control them, especially those that can lead to discrimination, stigmatization, harm, or injustice [89, 154, 188]. Indeed, bias reduction and management is essential to promoting public trust in AI (discussed in Sects. 5.5 and 5.7).

**Fig. 7:**
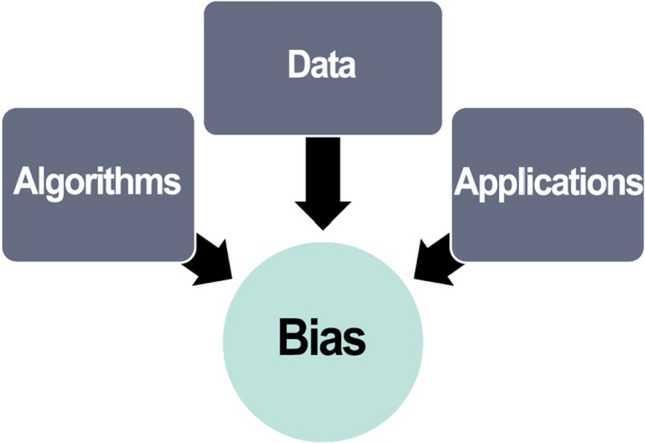
Sources of bias in AI/ML

Scientists have dealt with bias in research for years and have developed methods and strategies for minimizing and controlling bias in experimental design, data analysis, model building, and theory construction [79, 89, 104]. However, bias related to using AI in science can be subtle and difficult to detect due to the size and complexity of research data and interactions between data, algorithms, and applications [131]. See Fig. 7. Scientists who use AI systems in research should take appropriate steps to anticipate, identify, control, and minimize biases by ensuring that datasets reflect the diversity of the investigated phenomena and disclosing the variables, algorithms, models, and parameters used in data analysis [56]. Managing bias related to the use of AI should involve continuous testing of the outputs in real world applications and adjusting systems accordingly [70, 131]. For example, if a ML tool is used to read radiologic images, software developers, radiologists, and other stakeholders should continually evaluate the tool and its output to improve accuracy and precision.

### Random errors in AI

AI systems can make random errors even after extensive training [51, 151]. Nowhere has this problem been more apparent and concerning than in the use of LLMs in business, law, and scientific research. ChatGPT, for example, is prone to making random factual and citation errors. For example, Bhattacharyya et al. [24] used ChatGPT 3.5 to generate 30 short papers (200 words or less) on medical topics. 47% of the references produced by the chatbot were fabricated, 46% were authentic but inaccurately used, and only 7% were correct. Although ChatGPT 4.0 performs significantly better than ChatGPT 3.5, it still produces fabricated and inaccurate citations [230]. Another example of a random error was seen in a now-retracted paper published in *Frontiers in Cell Development and Biology*, which included an AI-generated image of a rat with unreal genitals [179]. Concerns raised by researchers led to OpenAI [173] warning users that “ChatGPT may produce inaccurate information about people, places, or facts.” The current interface includes the following disclaimer underneath the input box “ChatGPT can make mistakes. Consider checking important information.” Two US lawyers learned this lesson the hard way after a judge fined them $5000 for submitting court filing prepared by ChatGPT that included fake citations. The judge said that there was nothing improper about using ChatGPT but that the lawyers should exhibit due care in checking its work for accuracy [150].

An example of random errors made by generative AI discussed in the literature pertains to fake citations.^10^ One reason why LLM-based systems, such as ChatGPT produce fake, but realistic-looking citations is that they process text data differently from human beings. Researchers produce citations by reading a specific text and citating it, but ChatGPT *produces* citations by processing a huge amount of text data and generating a highly probable response to a request for a citation. Software developers at OpenAI, Google, and other chatbot companies have been trying to fix this problem, but it is not easy to solve, due to differences between human and LLM processing of language [24, 230]. AI companies advise users to use context-specific GPTs installed on top of ChatGPT. For instance, by using the Consensus.ai GPT (https://consensus.app/), which claims to be connected to “200M + scientific papers”, users can ask for specific citations for a given input (e.g., “coffee is good for human health”). While the offered citations are likely to be correct bibliometrically, errors and biases may not be fully removed because it is unclear how these systems come to their conclusions and offer specific citations (see discussion of the black box problem in Sect. 5.7).^11^

### Prospects for reducing AI errors

If AI systems follow the path taken by most other technologies, it is likely that errors will decrease over time as improvements are made [151]. For example, early versions of ChatGPT were very bad at solving math problems but newer versions are much better math because they include special GPTs for performing this task [210]. AI systems also make errors in reading, classifying, and reconstructing radiological images, but the error rate is decreasing, and AI systems will soon outperform humans in terms of speed and accuracy of image reading [12, 17, 103, 228]. However, it is also possible that AI systems will make different types of errors as they evolve or that there will be limits to their improvement. For example, newer versions of ChatGPT are prone to reasoning errors associated with intuitive thinking but older versions did not make these errors [91]. Also, studies have shown that LLMs are not good at self-correcting and need human supervision and fine-tuning to perform this task well [61].

Some types of errors may be difficult to eliminate due to differences between human perception/understanding and AI data processing. As discussed previously, AI systems, such as the system that generated the implausible hypothesis that laying down when having a radiologic image taken is a COVID-19 risk factor, make errors because they process information differently from humans. The AI system made this implausible inference because it did not factor basic biological and medical facts that would be obvious to doctors and scientists [170]. Another salient example of this phenomenon occurred when an image recognition AI was trained to distinguish between wolves and huskies, but it had difficulty recognizing huskies in the snow or wolves on the grass, because it had learned to distinguish between wolves and huskies by attending to the background of the images [222]. Humans are less prone to this kind of error because they use concepts to process perceptions and can therefore recognize objects in different settings. Consider, for example, captchas (Completely Automated Public Turing test to tell Computers and Humans Apart), which are used by many websites for security purposes and take advantage of some AI image processing deficiencies to authenticate whether a user is human [109]. Humans can pass Captchas tests because they learn to recognize images in various contexts and can apply what they know to novel situations [23].

Some of the factual and reasoning errors made by LLM-based systems occur because they lack human-like understanding of language [29, 135, 152, 153]. ChatGPT, for example, can perform well when it comes to processing language that has already been curated by humans, such as describing the organelles in a cell or explaining known facts about photosynthesis, but they may perform sub-optimally (and sometimes very badly) when dealing with novel text that requires reasoning and problem-solving because it does do not have a human-like understanding of language. When a person processes language, they usually form a mental model that provides meaning and context for the words [29]. This mental model is based on implicit facts and assumptions about the natural world, human psychology, society, and culture, or what we might call commonsense [119, 152, 153, 197]. LLMs do not do this,they only process symbols and predict the most likely string of symbols from linguistic prompts. Thus, to perform optimally, LLMs often need human supervision and input to provide the necessary meaning and context for language [61].

As discussed in Sect. 4, because AI systems do not process information in the way that humans do, it can be difficult to anticipate, understand and detect the errors these tools make. For this reason, continual monitoring of AI performance in real-world applications, including feedback from end-users, developers, and other stakeholders, is essential to AI quality control and quality improvement and public trust in AI [131, 174].

### Lack of moral agency

As mentioned in Sect. 2, narrow AI systems, such as LLMs, lack the capacities regarded as essential for moral agency, such as consciousness, self-concepts, personal memory, life experiences, goals, and emotions [18, 139, 151]. While this is not a problem for most technologies, it is for AI systems because they may be used to perform activities with significant moral and social consequences, such as reading radiological images or writing scientific papers (see discussion in Sect. 7.6), even though AI cannot be held morally or legally responsible or accountable. The lack of moral agency, when combined with other AI limitations, such as lack of a meaningful and human-like connection to the physical world, can produce dangerous results. For example, in 2021, Alexa, Amazon’s LLM-based voice-assistant, instructed a 10-year-old girl to stick a penny into an electric outlet when she asked it for a challenge to do [20]. In 2023, the widow of a Belgian man who committed suicide claimed that he had been depressed and was chatting with an LLM that encouraged him to kill himself [44, 69]). OpenAI and other companies have tried to put guardrails in place to prevent their systems from giving dangerous advice, but this is not easy to fix. A recent study found that while ChatGPT can pass medical boards, it can give dangerous medical advice due to its tendency to make factual errors and its lack of understanding of the meaning and context of language [51].

### The black box problem

**Fig. 8:**
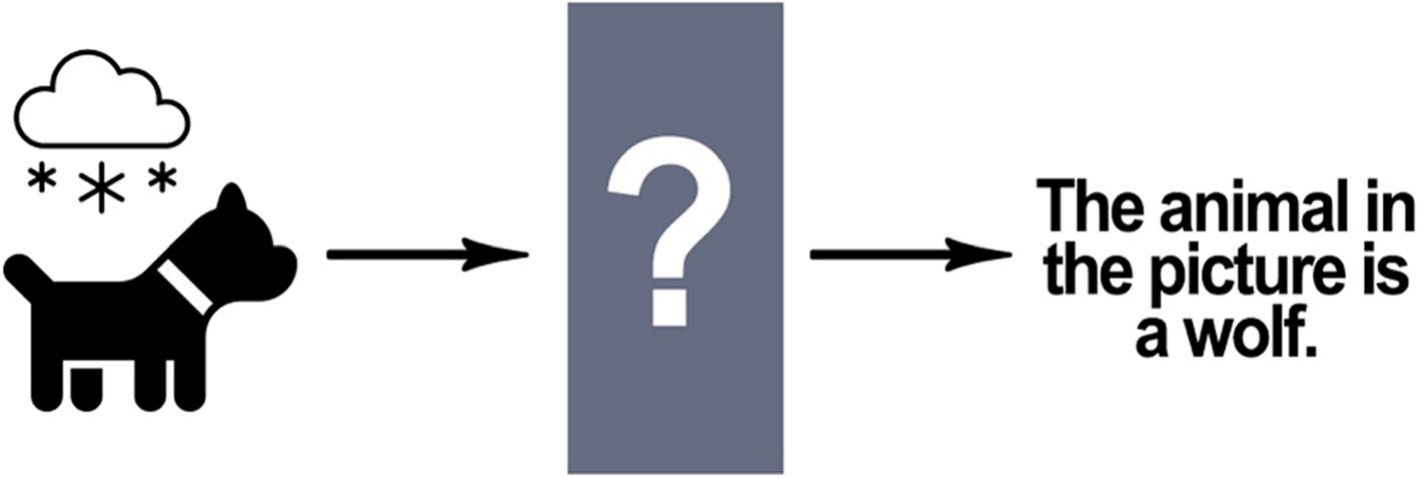
The black box: AI incorrectly labels a picture of a dog as a picture of a wolf but a complete investigation of this error is not possible due to a “black box” in the system

Suppose ChatGPT produces erroneous output, and a computer scientist or engineer wants to know why. As a first step, they could examine the training data and algorithms to determine the source of the problem.^12^ However, to fully understand what ChatGPT is doing they need to probe deeply into the system and examine not only the code but also the weightings attached to inputs in the ANN layers and the mathematical computations produced from the inputs. While an expert computer scientist or engineer could troubleshoot the code, they will not be able to interpret the thousands of numbers used in the weightings and the billions of calculations from those numbers [110, 151, 199]. This is what is meant when an AI system is described as a “black box.” See Fig. 8. Trying to understand the meaning of the weightings and calculations in ML is very different from trying to understand other types of computer programs, such as those used in most cell phones or personal computers, in which an expert could examine the system (as a whole) to determine what it is doing and why [151, 199].^13^

The opacity of AI systems is ethically problematic because one might argue that we should not use these devices if we cannot trust them, and we cannot trust them if even the best experts do not completely understand how they work [6, 7, 39, 47, 63, 186]. Trust in a technology is partially based on understanding that technology. If we do not understand how a telescope works, then we should not trust in what we see with it.^14^ Likewise, if computer experts do not completely understand how an AI/ML system works, then perhaps we should not use them for important tasks, such as making hiring decisions, diagnosing diseases, analyzing data, or generating scientific hypotheses or theories [63, 74].

The black box problem raises important ethical issues for science (discussed further in Sect. 7.4), because it can undermine public trust in science, which is already in decline, due primarily to the politicization of topics with significant social implications, such as climate change, COVID-19 vaccines and public health measures [123, 189].

One way of responding to the black box problem is to argue that we do not need to completely understand AI systems to trust them; what matters is an acceptably low rate of error [136, 186]. Proponents of this view draw an analogy between using AI systems and using other artifacts, such as using aspirin for pain relief, without fully understanding how they work. All that really matters for trusting a machine or tool is that we have evidence that it works well for our purposes, not that we completely understand how it works. This line of argument implies that it is justifiable to use AI systems to read radiological images, model the 3-D structures of proteins, or write scientific papers provided that we have evidence that they perform these tasks as well as human beings [136].

This response to the black box problem does not solve the problem but simply tells us not to worry about it [63].^15^ There are several reasons to be concerned about the black box problem. First, if something goes wrong with a tool or technology, regulatory agencies, injured parties, insurers, politicians, and others want to know precisely how it works to prevent similar problems in the future and hold people and organizations legally accountable [141]. For example, when the National Transportation Safety Board [160] investigates an airplane crash, they want to know what *precisely* went wrong. Was the crash due to human error? Bad weather? A design flaw? A defective part? The NTSB will not be satisfied with an explanation that appeals to a mysterious technology within the airplane.

Second, when regulatory agencies, such as the Food and Drug Administration (FDA), make decisions concerning the approval of new products, they want to know how the products work, so they can make well-informed, publicly-defendable decisions and inform the consumers about risks. To obtain FDA approval for a new drug, a manufacturer must submit a vast amount of information to the agency, including information about the drug’s chemistry, pharmacology, and toxicology; the results of pre-clinical and clinical trials; processes for manufacturing the drug; and proposed labelling and advice to healthcare providers [75]. Indeed, dealing with the black box problem has been a key issue in FDA approval of medical devices that use AI/ML [74, 183].

Third, end-users of technologies, such as consumers, professionals, researchers, government officials, and business leaders may not be satisfied with black boxes. Although most laypeople comfortably use technologies without fully understanding their innerworkings, they usually assume that experts who understand how these technologies work have assessed them and deemed them to be safe. End-users may become highly dissatisfied with a technology when it fails to perform its function, especially when not even the experts can explain why. Public dissatisfaction with responses to the black box problem may undermine the adoption of AI/ML technologies, especially when these technologies cause harm, invade privacy, or produce biased claims and results [60, 85, 134, 175].

### Explainable AI

An alternative to the non-solution approach is to make AI explainable [11, 96, 151, 186]. The basic idea behind explainability is to develop “processes and methods that allows human users to comprehend and trust the results and output created by machine learning algorithms” [110]. Transparency of algorithms, models, parameters, and data is essential to making AI explainable, so that users can understand an AI system’s accuracy and precision and the types of errors it is prone to making. Explainable AI does not attempt to “peer inside” the black box, but it can make AI behavior more understandable to developers, users, and other stakeholders. Explainability, according to proponents of this approach, helps to promote trust in AI because it allows users and other stakeholders to make rational and informed decisions about it [77, 83, 110, 186].

While the explainable AI approach is preferable to the non-solution approach, it still has some shortcomings. First, it is unclear whether making AI explainable will satisfy non-experts because considerable expertise in computer science and/or data analytics may be required to understand what is being explained [120, 186]. For transparency to be effective, it must address the audience’s informational needs [68]. Explainable AI, at least in its current form, may not address the informational needs of laypeople, politicians, professionals, or scientists because the information is too technical [58]. To be explainable to non-experts, the information should be expressed in plain, jargon-free language that describes what the AI did and why [96].

Second, it is unclear whether explainable AI completely solves issues related to accountability and legal liability because we are yet to witness how legal systems will deal with AI lawsuits in which information pertaining to explainability (or lack thereof) is used as evidence in a court [141]. However, it is conceivable that the information conveyed to make AI explainable will satisfy the courts in some cases and set judicial precedent, so that legal doctrines and practices related to liability for AI-caused harms will emerge, much in the same way that doctrines and practices for medical technologies emerged.

Third, there is also the issue of whether explainable AI will satisfy the requirements of regulatory agencies, such as the FDA. However, regulatory agencies have been making some progress toward addressing the black box problem and explainability is likely to play a key role in these efforts [183].

Fourth, private companies uninterested in sharing information about their systems may not comply with explainable AI requirements or they may “game” the requirements to resemble compliance without actually complying. ChatGPT, for example, is a highly opaque system that is yet to disclose its training data and it is unclear whether/when OpenAI would open up its technology to external scrutiny [28, 66, 130].

Despite these shortcomings, the explainable AI approach is a reasonable way of dealing with transparency issues, and we encourage its continued development and application to AI/ML systems.

## Ethical norms of science

With this overview of AI in mind, we can now consider how using AI in research impacts the ethical norms of science. But first, we need to describe these norms. Ethical norms of science are principles, values, or virtues that are essential for conducting good research [147, 180, 187, 191]. See Table 1. These norms apply to various practices, including research design; experimentation and testing; modelling; concept formation; data collection and storage; data analysis and interpretation; data sharing; publication; peer review; hypothesis/theory formulation and acceptance; communication with the public; as well as mentoring and education [207]. Many of these norms are expressed in codes of conduct, professional guidelines, institutional or journal policies, or books and papers on scientific methodology [4, 10, 113, 235]. Others, like collegiality, might not be codified but are implicit in the practice of science. Some norms, such as testability, rigor, and reproducibility, are primarily epistemic, while others, such as fair attribution of credit, protection of research subjects, and social responsibility, are primarily moral (when enshrined in law, like instance of fraud, these norms become legal but here we only focus on ethical norms). There are also some like honesty, openness, and transparency, which have both epistemic and moral dimensions [191, 192].

**Table 1:** Norms of science (based on [191, 192])

Honesty	Accountability
Testability	Freedom of inquiry
Rigor	Fair sharing of credit
Empiricism	Confidentiality of peer review
Skepticism	Collegiality
Explanatory power	Non-discrimination
Objectivity	Respect for intellectual property
Realism	Protection of human subjects
Precision	Protection of animal subjects
Openness	Safety (physical, biological, psychosocial)
Transparency	Stewardship of resources
Reproducibility	Social responsibility

These are some commonly discussed norms. We do not claim that our list is original, exhaustive, or exclusive

Scholars from different fields, including philosophy, sociology, history, logic, decision theory, and statistics have studied ethical norms of science [84, 89, 104, 125, 128, 137, 147, 180, 208, 209, 237]. Sociologists such as Merton [147] and Shapin [208], tend to view ethical norms as generalizations that accurately describe the practice of science, while philosophers, such as Kitcher [125] and Haack [89], conceive of these norms as prescriptive standards that scientists *ought* to follow. These approaches need not be mutually exclusive, and both can offer useful insights about ethical norms of science. Clearly, the study of norms must take the practice of science as its starting point, otherwise our understanding of norms would have no factual basis. However, one cannot simply infer the ethical norms of science from the practice of science because scientists may endorse and defend norms without always following them. For example, most scientists would agree that they should report data honestly, disclose significant conflicting interests, and keep good research records, but evidence indicates that they sometimes fail to do so [140].

One way of bridging the gap between descriptive and prescriptive accounts of ethical norms of science is to reflect on the social and epistemological foundations (or justifications) of these norms. Ethical norms of science can be justified in at least three ways [191].

First, these norms help the scientific community achieve its epistemic and practical goals, such as understanding, predicting, and controlling nature. It is nearly impossible to understand how a natural or social process works or make accurate predictions about it without standards pertaining to honesty, logical consistency, empirical support, and reproducibility of data and results. These and other epistemic standards distinguish science form superstition, pseudoscience, and sophistry [89].

Second, ethical norms promote trust among scientists, which is essential for collaboration, peer review, publication, sharing of data and resources, mentoring, education, and other scientific activities. Scientists need to be able to trust that the data and results reported in papers have not been fabricated, falsified, or manipulated; that reviewers for journals and funding agencies will maintain confidentiality; that colleagues or mentors will not steal their ideas and other forms of intellectual property; and that credit for collaborative work will be distributed fairly [26, 233].

Third, ethical norms are important for fostering public support for science. The public is not likely to financially, legally, or socially support research that is perceived as corrupt, incompetent, untrustworthy, or unethical [191]. Taken together, these three modes of justification link ethical norms to science’s social foundations; that is, ethical norms are standards that govern the scientific community, which itself operates within and interacts with a larger community, namely society [137, 187, 209].

Although vital for conducting science, ethical norms are not rigid rules. Norms sometimes conflict, and when they do, scientists must make decisions concerning epistemic or moral priorities [191]. For example, model-building in science may involve tradeoffs among various epistemic norms, including generality, precision, realism, simplicity, and explanatory power [143]. Research with human subjects often involves tradeoffs between rigor and protection of participants. For example, placebo control groups are not used in clinical trials when receiving a placebo instead of an effective treatment would cause serious harm to the participant [207].

Although the norms can be understood as guidelines, some have higher priority than others. For example, honesty is the hallmark of good science, and there are very few situations in which scientists are justified in deviating from this norm.^16^ Openness, on the other hand, can be deemphasized to protect research participants’ privacy, intellectual property, classified information, or unpublished research [207].

Finally, science’s ethical norms have changed over time, and they are likely to continue to evolve [80, 128, 147, 237]. While norms such as empiricism, objectivity, and consistency originated in ancient Greek science, others, such as reproducibility and openness, developed during the 1500s; and many, such as protection of research subjects and social responsibility, did not emerge as formalized norms until the twentieth century. This evolution is in response to changes in science’s social, institutional, economic, and political environment and advancements in scientific instruments, tools, and methods [100]. For example, the funding of science by private companies and their requirements concerning data access and release policies have led to changes in norms related to open sharing of data and materials [188]. The increased presence of women and racial and ethnic minorities in science has led to the development of policies for preventing sexual and other forms of harassment [185]. The use of computer software to analyze large sets of complex data has challenged traditional views about norms related to hypothesis testing [193, 194].

## AI and the ethical norms of science

We will divide our discussion of AI and the ethics of science into six topics corresponding to the problems and issues previously identified in this paper and seventh topic related to scientific education. While these topics may seem somewhat disconnected, they all involve ethical issues that scientists who use AI in research are currently dealing with.

### AI biases and the ethical norms of science

Bias can undermine the quality and trustworthiness of science and its social impacts [207]. While reducing and managing bias are widely recognized as essential to good scientific methodology and practice [79, 89], they become crucial when AI is employed in research because AI can reproduce and amplify biases inherent in the data and generate results that lend support to policies that are discriminatory, unfair, harmful, or ineffective [16, 202]. Moreover, by taking machines’ disinterestedness in findings as a necessary and sufficient condition of objectivity, users of AI in research may overestimate the objectivity of their findings. AI biases in medical research have generated considerable concern, since biases related to race, ethnicity, gender, sexuality, age, nationality, and socioeconomic status in health-related datasets can perpetuate health disparities by supporting biased hypotheses, models, theories, and policies [177, 198, 211]. Biases also negatively impact areas of science outside the health sphere, including ecology, forestry, urban planning, economics, wildlife management, geography, and agriculture [142, 164, 165].

OpenAI, Google, and other generative AI developers have been using filters that prevent their systems from generating text that is outright racist, sexist, homophobic, pornographic, offensive, or dangerous [93]. While bias reduction is a necessary step to make AI safe for human use, there are reasons to be skeptical of the idea that AI can be appropriately sanitized. First, the biases inherent in data are so pervasive that no amount of filtering can remove all of them [44, 69]. Second, AI systems may also have political and social biases that are difficult to identify or control [19]. Even in the case of generative AI models where some filtering has happened, changing the inputted prompt may simply confuse and push a system to generate biased content anyway [98].

Third, by removing, reducing and controlling some biases, AI developers may create other biases, which are difficult to anticipate, identify or describe at this point. For example, LLMs have been trained using data gleaned from the Internet, scholarly articles and Wikipedia [90], all of which consist of the broad spectrum of human behavior and experience, from good to bad and virtuous to sinister. If we try to weed undesirable features of this data, we will eliminate parts of our language and culture, and ultimately, parts of us.^17^ If we want to use LLMs to make sound moral and political judgments, sanitizing their data processing and output may hinder their ability to excel at this task, because the ability to make sound moral judgements or anticipate harm may depend, in part, on some familiarity with immoral choices and the darker side of humanity. It is only by understanding evil that we can freely and rationally choose the good [40]. We admit this last point is highly speculative, but it is worth considering. Clearly, the effects of LLM bias-management bear watching.

While the problem of AI bias does not require a radical revision of scientific norms, it does imply that scientists who use AI systems in research have special obligations to identify, describe, reduce, and control bias [132]. To fulfill these obligations, scientists must not only attend to matters of research design, data analysis and interpretation, but also address issues related to data diversity, sampling, and representativeness [70]. They must also realize that they are ultimately accountable for AI biases, both to other scientists and to members of the public. As such, they should only use AI in contexts where their expertise and judgement are sufficient to identify and remove biases [97]. This is important because given the accessibility of AI systems and the fact that they can exploit our cognitive shortcomings, they are creating an illusion of understanding [148].

Furthermore, to build public trust in AI and promote transparency and accountability, scientists who use AI should engage with impacted populations, communities and other stakeholders to address their needs and concerns and seek their assistance in identifying and reducing potential biases [132, 181, 202].^18^ During the engagement process, researchers should help populations and communities understand how their AI system works, why they are using it, and how it may produce bias. To address the problem of AI bias, the Biden Administration recently signed an executive order that directs federal agencies to identify and reduce bias and protect the public from algorithmic discrimination [217].

### AI random errors and the ethical norms of science

Like bias, random errors can undermine the validity and reliability of scientific knowledge and have disastrous consequences for public health, safety, and social policy [207]. For example, random errors in the processing of radiologic images in a clinical trial of a new cancer drug could harm patients in the trial and future patients who take an approved drug, and errors related to the modeling of the transmission of an infectious disease could undermine efforts to control an epidemic. Although some random errors are unavoidable in science, an excessive amount when using AI could be considered carelessness or recklessness when using AI (see discussion of misconduct in Sect. 7.3).

Reduction of random errors, like reduction of bias, is widely recognized as essential to good scientific methodology and practice [207]. Although some random errors are unavoidable in research, scientists have obligations to identify, describe, reduce, and correct them because they are ultimately accountable for both human and AI errors. Scientists who use AI in their research should disclose and discuss potential limitations and (known) AI-related errors. Transparency about these is important for making research trustworthy and reproducible [16].

Strategies for reducing errors in science include time-honored quality assurance and quality improvement techniques, such as auditing data, instruments, and systems; validating and testing instruments that analyze or process data; and investigating and analyzing errors [1]. Replication of results by independent researchers, journal peer review, and post-publication peer review also play a major role in error reduction [207]. However, given that content generated by AI systems is not always reproducible [98], identifying and adopting measures to reduce errors is extremely complicated. Either way, accountability requires that scientists take responsibility for errors produced by AI/ML systems, that they can explain why errors have occurred, and that they transparently share their limitations of their knowledge related to these errors.

### AI and research misconduct

Failure to appropriately control AI-related errors could make scientists liable for research misconduct, if they intentionally, knowingly, or recklessly disseminate false data or plagiarize [207].^19^ Although most misconduct regulations and policies distinguish between misconduct and honest error, scientists may still be liable for misconduct due to recklessness [42, 193, 194], which may have consequences for using AI.^20^ For example, a person who uses ChatGPT to write a paper without carefully checking its output for errors or plagiarism could be liable for research misconduct for reckless use of AI. Potential liability for misconduct is yet another reason why using AI in research requires taking appropriate steps to minimize and control errors.

It is also possible that some scientists will use AI to fabricate data or images presented in scientific papers, grant proposals, or other documents. This unethical use of AI is becoming increasingly likely since generative models can be used to create synthetic datasets from scratch or make alternative versions of existing datasets [50, 155, 200, 214]. Synthetic data are playing an increasingly important role in some areas of science. For example, researchers can use synthetic data to develop and validate models and enhance statistical analysis. Also, because synthetic data are similar to but not the same as real data, they can be used to eliminate or mask personal identifiers and protect the confidentiality of human participants [31, 81, 200].

Although we do not know of any cases where scientists have been charged with research misconduct for presenting synthetic data as real data, it is only a matter of time until this happens, given the pressures to produce results, publish, and obtain grants, and the temptations to cheat or cut corners.^21^ This speculation is further corroborated by the fact that a small proportion of scientists deliberately fabricate or falsify data at some point in their careers [73, 140]. Also, using synthetic data in research, even appropriately, may blur the line between real and fake data and undermine data integrity. Researchers who use synthetic data should (1) indicate which parts of data are synthetic, (2) describe how the data were generated; (3) explain how and why they were used [221].

### The black box problem and the ethical norms of science

The black box problem presents significant challenges to the trustworthiness and transparency of research that use AI because some of the steps in the scientific process will not be fully open and understandable to humans, including AI experts. An important implication of the black box problem is that scientists who use AI are obligated to make their use of the technology explainable to their peers and the public. While precise details concerning what makes an AI system explainable may vary across disciplines and contexts, some baseline requirements for transparency may include:

- The type, name, and version of AI system used.
- What task(s) the system was used for.
- How, when and by which contributor a system was used.
- Why a certain system was used instead of alternatives (if available).
- What aspects of a system are not explainable (e.g., weightings).
- Technical details related to model’s architecture, training data and optimization procedures, influential features involved in model’s decisions, the reliability and accuracy of the system (if known).
- Whether inferences drawn by the AI system are supported by currently accepted scientific theories, principles, or concepts.

This information should be expressed in plain language to allow non-experts to understand the whos, whats, hows, and whys related to the AI system. Ideally, this information would become a standard part of reported research that used AI. The information could be reported in the materials and methods section or in supplemental material, much that same way that information about statistical methods and software is currently reported.

As mentioned previously, making AI explainable does not completely solve the black box problem but it can play a key role in promoting transparency, accountability, and trust [7, 9]. While there seems to be an emerging consensus on the utility and importance of making AI explainable, there is very little agreement about what explainability means in practice, because what makes AI explainable depends on the context of its use [58]. Clearly, this is a topic where more empirical research and ethical/policy analysis is needed.

### AI and confidentiality

Using AI in research, especially generative AI models, raises ethical issues related to data privacy and confidentiality. ChatGPT, for example, stores the information submitted by users, including data submitted in initial prompts and subsequent interactions. Unless users opt out, this information could be used for training and other purposes. The data could potentially include personal and confidential information, such as information contained in drafts of scientific papers, grant proposals, experimental protocols, or institutional policies; computer code; legal strategies; business plans; and private information about human research participants [67, 85]. Due to concerns about breaches of confidentiality, the National Institutes of Health (NIH) recently prohibited the use of generative AI technologies, such as LLMs, in grant peer review [159].^22^ Some US courts now require lawyers to disclose their use of generative AI in preparing legal documents and make assurances that they have taken appropriate steps to protect confidentiality [146].

While we are not suggesting that concerns about confidentiality justify prohibiting generative AI use in science, we think that considerable caution is warranted. Researchers who use generative AI to edit or review a document should assume that the material contained in it will not be kept confidential, and therefore, should not use these systems to edit or review anything containing confidential or personal information.

It is worth noting that technological solutions to the confidentiality problem may be developed in due course. For example, if an organization operates a local application of an LLM and places the technology behind a secure firewall, its members can use the technology safely. Electronic medical records, for example, have this type of security [127]. Some universities have already begun experimenting with operating their own AI systems for use by students, faculty, and administrators [225]. Also, as mentioned in Sect. 7.3, the use of synthetic data may help to protect confidentiality.

### AI and moral agency

The next issue we will discuss is whether AI can be considered a moral agent that participates in an epistemic community, that is, as a partner in knowledge generation. This became a major issue for the ethical norms of science in the winter of 2022–2023, when some researchers listed ChatGPT as authors on papers [102]. These publications initiated a vigorous debate in the research community, and journals scrambled to develop policies to deal with LLMs’ use in research. On one end of the spectrum, Jenkins and Lin [116] argued that AI systems can be authors if they make a substantial contribution to the research, and on the other end, Thorp [218] argued that AI systems cannot be named as authors and should not be used at all in preparing manuscripts. Currently, there seems to be an emerging consensus that falls in between these two extremes position, namely, that AI systems can be used in preparing manuscripts but that their use should be appropriately disclosed and discussed, [4, 102]. In 2023, the International Committee of Medical Journal Editors (ICMJE), a highly influential organization with over 4,500 member journals, released the following statement about AI and authorship:

At submission, the journal should require authors to disclose whether they used artificial intelligence (AI)assisted technologies (such as Large Language Models [LLMs], chatbots, or image creators) in the production of submitted work. Authors who use such technology should describe, in both the cover letter and the submitted work, how they used it. Chatbots (such as ChatGPT) should not be listed as authors because they cannot be responsible for the accuracy, integrity, and originality of the work, and these responsibilities are required for authorship (see Section II.A.1). Therefore, humans are responsible for any submitted material that included the use of AI-assisted technologies. Authors should carefully review and edit the result because AI can generate authoritative-sounding output that can be incorrect, incomplete, or biased. Authors should not list AI and AI assisted technologies as an author or co-author, nor cite AI as an author. Authors should be able to assert that there is no plagiarism in their paper, including in text and images produced by the AI. Humans must ensure there is appropriate attribution of all quoted material, including full citations [113].

We agree with the ICMJE’s position, which mirrors views we defended in print before the ICMJE released its guidance [101, 102].

Authorship on scientific papers is based not only on making a substantial contribution, but also on being accountable for the work [207]. Because authorship implies significant epistemic and ethical responsibilities, one should not be named as an author on a work if one cannot be accountable for one’s contribution to the work. If questions arise about the work after publication, one needs to be able to answer those questions intelligibly and if deemed liable, face possible legal, financial, or social consequences for one’s actions.

AI systems cannot be held accountable for their actions for two reasons: (1) they cannot provide intelligible explanations for what they did, (2) they cannot be held morally responsible for their actions, (3) they cannot suffer consequences nor can be sanctioned. The first reason has to do with the previously discussed black box problem. Although current proposals for making AI explainable may help to deal with this issue, they still fall far short of humanlike accountability, because these proposals do not require that the AI system, itself, should provide an explanation. Regarding the second reason, when we hold humans accountable, we expect them to explain their behavior in clear and intelligible language.^23^ If a principal investigator wonders why a graduate student did not report all the data related to experiment, the investigator expects the student to explain why they did what they did. Current AI systems cannot do this. In some cases, someone else may be able to provide an explanation of how they work and what they do, but this not the same as the AI providing the explanation, which is a prerequisite for accountability. The third reason has to do with the link between accountabilities and sanctions. If an AI system makes a mistake which harms others, it cannot be sanctioned. These systems do not have interests, values, reputation and feelings in the same way that humans do and cannot be punished by law enforcement.

Even if an AI can intelligibly explain itself in the future, this does not imply that it can be morally responsible. While the concept of moral agency, like the concept of consciousness, is controversial, there is general agreement that moral agency requires the capacity to perform intentional (or purposeful) actions, understand moral norms, and make decisions based on moral norms. These capacities also presuppose additional capacities, such as consciousness, self-awareness, personal memory, perception, general intelligence, and emotions [46, 95, 213]. While computer scientists are making some progress on developing AI systems that have quasi-moral agency, that is, AI systems that can make decisions based on moral norms [71, 196, 203], they are still a long way from developing AGI or AC (see definitions of these terms in Sect. 2), which would seem to be required for genuine moral agency.

Moreover, other important implications follow from current AI’s lack of moral agency. First, AI systems cannot be named as inventors on patents, because inventorship also implies moral agency [62]. Patents are granted to individuals, i.e., persons, but since AI systems lack moral agency, they do not qualify as persons under the patent laws adopted by most countries. Second, AI systems cannot be copyright holders, because to own a copyright, one must be a person [49]. Copyrights, under US law, are granted only to people [224].

Although AI systems should not be named as authors or inventors, it is still important to appropriately recognize their contributions. Recognition should be granted not only to promote honesty and transparency in research but also to prevent human authors from receiving undue credit. For example, although many scientists and engineers deserve considerable accolades for solving the protein folding problem [118, 176], failing to mention the role of AlphaFold in this discovery would be giving human contributors more credit than they deserve.

### AI and research ethics education

The last topic we will address in this section has to do with education and mentoring in responsible conduct of research (RCR), which is widely recognized as essential to promoting ethical judgment, reasoning, and behavior in science [207]. In the US, the NIH and National Science Foundation (NSF) require RCR education for funded students and trainees, and many academic institutions require some form of RCR training for all research faculty [190]. Topics typically covered in RCR courses, seminars, workshops, or training sessions include data fabrication and falsification, plagiarism, investigation of misconduct, scientific record keeping, data management, rigor and reproducibility, authorship, peer review, publication, conflict of interest, mentoring, safe research environment, protection and human and animal subjects, and social responsibility [207]. As demonstrated in this paper, the use of AI in research has a direct bearing on most of these topics, but especially on authorship, rigor and reproducibility, peer review, and social responsibility. We recommend, therefore, that RCR education and training incorporate discussion of the use of AI in research, wherever relevant.

## Conclusion

Using AI in research benefits science and society but also creates some novel and complex ethical issues that affect accountability, responsibility, transparency, trustworthiness, reproducibility, fairness, and objectivity, and other important values in research. Although scientists do not need to radically revise their ethical norms to deal with these issues, they do need new guidance for the appropriate use of AI in research. Table 2 provides a summary of our recommendations for this guidance. Since AI continues to advance rapidly, scientists, academic institutions, funding agencies and publishers, should continue to discuss AI’s impact on research and update their knowledge, ethical guidelines and policies accordingly. Guidance should be periodically revised as AI becomes woven into the fabric of scientific practice (or normalized) and researchers learn about it, adapt to it, and use it in novel ways. Since science has significant impacts on society, public engagement in such discussions is crucial for responsible the use, development, and AI in research [234].

**Table 2:** Recommendations for Ethical Use of AI in Research

Recommendation	Normative justification
Researchers are responsible for identifying, describing, reducing, and controlling AI-related biases and random errors	Accountability, objectivity, reproducibility, rigor, transparency, honesty, social responsibility, fairness
Researchers should disclose, describe, and explain their use of AI in research, including its limitations, in language that can be understood by non-experts	Accountability, transparency, reproducibility, rigor, objectivity, social responsibility, fairness
Researchers should use AI only in situations in which they have sufficient expertise or judgement to use it responsibly	Accountability, carefulness, reproducibility, social responsibility, rigor, objectivity
Researchers should engage with impacted communities, populations, and other stakeholders concerning the use of AI in research to obtain their advice and assistance and address their interests and concerns, such as issues related to bias	Accountability, transparency, social responsibility, rigor, fairness
Researchers who intentionally, knowingly, or recklessly use AI to fabricate or falsify data or commit plagiarism are liable for misconduct	Accountability, honesty, reproducibility, rigor, legality
Researchers who use synthetic data should (1) indicate which parts of the data are synthetic; (2) clearly label the synthetic data; (3) describe how the data were generated; and (4) explain how and why the data were used	Accountability, transparency, objectivity, honesty, reproducibility, rigor
AI systems should not be named as authors, inventors, or copyright holders but their contributions to research should be disclosed and described	Honesty, transparency, accountability, fair attribution of credit, legality
AI systems should not be used in situations that are likely to involve unauthorized disclosure of confidential information related to human research subjects, unpublished research, potential intellectual property claims, or proprietary or classified research	Protection of and respect for human subjects, legality, confidentiality of peer review, social responsibility, fairness
Education and mentoring in responsible conduct of research should include discussion of ethical use of AI	Accountability, reproducibility, rigor, social responsibility, honesty, transparency, fair attribution of credit

In closing, we will observe that many scholars, including ourselves, assume that today’s AI systems lack the capacities necessary for moral agency. This assumption has played a key role in our analysis of ethical uses of AI in research and has informed our recommendations. We realize that a day may arrive, possibly sooner than many would like to believe, when AI will advance to the point that this assumption will need to be revised, and that society will need to come to terms with the moral rights and responsibilities of some types of AI systems. Perhaps AI systems will one day participate in science as *full partners* in discovery and innovation [33, 126]. Although we do not view this as a matter that now demands immediate attention, we remain open to further discussion of this issue in the future.

## Footnotes

1 There is not sufficient space in this paper to conduct a thorough review of all the ways that AI is being used in scientific research. For a review of the information, see Wang et al. [231] and Krenn et al. [126].

2 However, the National Institutes of Health has prohibited the use of AI to review grants (see Sect. 7.5).

3 This a simplified taxonomy of AI that we have found useful to frame the research ethics issues. For more detailed taxonomy, see Graziani et al. [86].

4 See Krenn et al. [126] for a thoughtful discussion of the possible role of AGI in scientific research.

5 We will use the term ‘input’ in a very general sense to refer to data which are routed into the system, such as numbers, text, or image pixels.

6 It is important to note that the [167] paper was corrected to remove ChatGPT as an author because the tool did not meet the journal’s authorship criteria. See O’Connor [166].

7 There are important, philosophical issues at stake here concerning whether AI users *should* regard an output as ‘acceptable’ or ‘true’, but these questions are beyond the scope of our paper.

8 The question of whether true randomness exists in nature is metaphysically controversial because some physicists and philosophers argue that nothing happens by pure chance [64]. We do not need to delve into this issue here, since most people agree that the distinction can be viewed as epistemic and not metaphysical, that is, an error is systemic or random relative to our knowledge about the generation of the error.

9 Some of the most well-known cases of bias involved the use of AI systems by private companies. For example, Amazon stopped using an AI hiring tool in 2018 after it discovered that the tool was biased against women [57]. In 2021, Facebook faced public ridicule and shame for using image recognition software the labelled images of African American men as non-human primates [117]. In 2021, Zillow lost hundreds of millions of dollars because its algorithm systematically overestimated the market value of homes the company purchased [170].

10 Fake citations and factual errors made by LLMs are often referred to as ‘hallucinations.’ We prefer not to use this term because it ascribes mental states to AI.

11 An additional, and perhaps more concerning, issue is that using chatbots to review the literature contributes to the deskilling of humanity because it involves trusting an AI’s interpretation and synthesis of the literature instead of reading it and thinking about it for oneself. Since deskilling is a problem with many different applications of AI, we will not explore it in depth here. See Vallor [226].

12 We are assuming here that the engineer or scientist has access to the computer code and training data, which private companies may to be loath to provide. For example, developers at OpenAI and Google have not provided the public with access to their training data and code [130].

13 Although our discussion of the black box problem focuses on ML, in theory this problem could arise in any type of AI in which its workings cannot be understood by human beings.

14 Galileo had to convince his critics that his telescope could be trusted to convey reliable information about heavenly bodies, such as the moon and Jupiter. Explaining how the telescope works and comparing it to the human eye played an important role in his defense of the instrument [36].

15 This response may also conflate trust with verification. According to some theories of trust, if you trust something, you do not need to continually verify it. If I trust someone to tell me the truth, I do not need to continually verify that they are telling the trust. Indeed, it seems that we verify because we do not trust. For further discussion, see McLeod [145].

16 One could argue that deviation from honesty might be justified to protect human research subjects in some situations. For example, pseudonyms are often used in qualitative social/behavioral research to refer to participants or communities in order to protect their privacy [92].

17 Sanitizing LLMs is a form of censorship, which may be necessary in some cases, but also carries significant risks for freedom of expression [236].

18 While public, community, and stakeholder engagement is widely accepted as important for promoting trust in science and technology but can be difficult to implement, especially since publics, communities, and stakeholders can be difficult to identify and may have conflicting interests [157].

19 US federal policy defines research misconduct as data fabrication or falsification or plagiarism [168].

20 While the difference between recklessness and negligence can be difficult to ascertain, one way of thinking of recklessness is that it involves an indifference to or disregard for the veracity or integrity of research. Although almost all misconduct findings claim that the accused person (or respondent) acted intentionally, knowingly, or recklessly, there have been a few cases in which the respondent was found only to have acted recklessly [42, 193, 194].

21 The distinction between synthetic and real data raises some interesting and important philosophical and policy issues that we will examine in more depth in future work.

22 Some editors and publishers have been using AI to review and screen journal submissions [35, 212]. For a discussion of issues raised by using AI in peer review, see Hosseini and Horbach [98, 99].

23 This issue reminds us of the scene in *2001: A Space Odyssey* in which the human astronauts ask the ship’s computer, HAL, to explain why it incorrectly diagnosed a problem with the AE-35 unit. HAL responds that HAL 9000 computers have never made an error so the misdiagnosis must be due to human error.
